# Coupled Cluster Free Energies for Atmospheric Molecular Clusters: Benchmark and Matching Experimental Free Energies

**DOI:** 10.1021/acsomega.6c00573

**Published:** 2026-06-17

**Authors:** Yosef Knattrup, Andreas Buchgraitz Jensen, Jonas Elm

**Affiliations:** Department of Chemistry, Aarhus University, Langelandsgade 140, Aarhus C 8000, Denmark

## Abstract

The initial formation of secondary aerosols, a large cause of uncertainty in modern radiative forcing modeling, can be simulated using quantum chemical methods. When based on quantum chemistry, the simulations have an exponential dependence on the free energy, requiring a high-accuracy description. In this study, we have computed harmonic frequencies and quasi-harmonic free energies for a set of 11 monomers and 27 dimers relevant for atmospheric molecular clusters at the DF-CCSD­(F12b)­(T*)/cc-pVDZ-F12 level of theory. The set is used to benchmark the M06-2X, PW91, ωB97X-D3BJ functional with the Jensen, Karlsruhe, and Pople style basis sets. The composite methods B97-3c, r^2^SCAN-3c, and ωB97X-3c are also tested. We find ωB97X-D3BJ/ma-def2-SVP to be an optimal choice, as it has low errors (mean absolute error of 0.13 kcal/mol) and few outliers in the thermal contribution. For calculations on larger clusters, B97-3c stands out. Using the ωB97X-D3BJ/ma-def2-SVP and B97-3c level of theories, we compute anharmonic frequencies using the VPT2 method and determine an anharmonic scaling factor of 0.961 and 0.953 for ωB97X-D3BJ/ma-def2-SVP and B97-3c frequencies, respectively. The scaling factor together with incorporating multiconformer entropy effects and a high-level single-point correction at the Normal LNO-CCSD­(T)/CBS­(aug-3,aug-4) are used to compare to experimentally determined free energies of the 11 hydrogen-bonded systems. We find that we obtain sub 1 kcal/mol errors when incorporating the scaling factor, the multiconformer entropy effects, and the single point correction.

## Graphical abstract

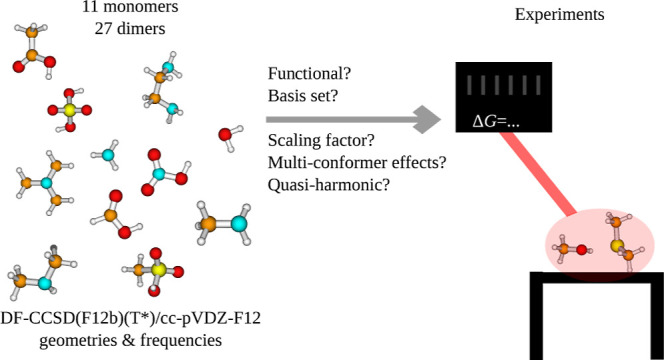

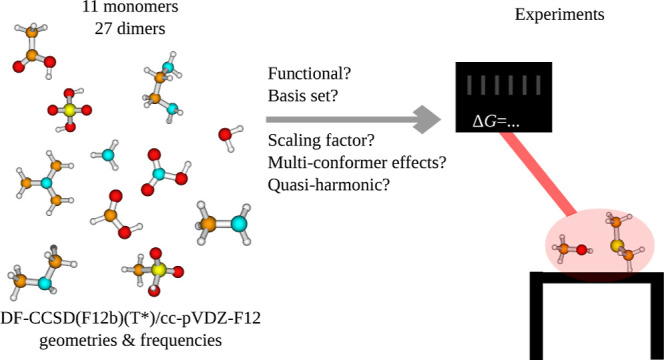

## 1 Introduction

According to the sixth assessment report of the Intergovernmental Panel on Climate Change, aerosols (liquids or solids suspended in air) remain the largest source of uncertainty in current global climate models due to their effects on radiative forcing.^1^ Aerosols influence climate through direct interaction with sunlight via scattering and absorption, and indirectly by serving as cloud condensation nuclei (CCN) for cloud droplet formation.^2,3^ Of these mechanisms, the uncertainty in CCN formation has the greatest impact on modeling accuracy, the uncertainty primarily stems from incomplete understanding of the initial formation steps of the particles.^4^ New particle formation (NPF), where atmospheric gas-phase precursors undergo gas-to-particle conversions, accounts for approximately 60% of CCN over over Western United States land and 30–40% over mainland Europe.^5,6^ This process is predominantly driven by the clustering of sulfuric acid (SA) with bases such as ammonia (AM)^5,7–11^ and alkyl-amines including methylamine (MA), dimethylamine (DMA),^10,12–21^ and trimethylamine (TMA),^10,14,16,17,19,20^ with additional enhancement from acids like nitric acid (NA),^22–33^ formic acid (FA),^30,32,34–38^ methanesulfonic acid (MSA),^39–47^ and acetic acid (AA)^38,48–51^ or water (W).^15,52–66^

NPF rates can be modeled using cluster distribution dynamics simulations,^67^ where birth–death equations describe the evolution of cluster concentrations through collision and evaporation events. The evaporation coefficient,^68^ depends exponentially on the cluster binding free energies through detailed mass balance, requiring high accuracy in the free energy calculations to avoid exponentially growing errors in predicted NPF rates. Previous studies have established that the electronic energy is the largest source of error in binding free energy calculations.^69,70^ We have shown that using the LNO–CCSD­(T) method^71–76^ with a complete basis set extrapolation yields a mean absolute error (MAE) within 0.2 kcal/mol of the CCSD­(F12*)­(T+)/cc-pVTZ-F12 reference for smaller atmospheric molecular clusters, and the method is applicable to clusters with 300 atoms.^77^

However, the total binding free energy comprises both electronic and thermal contributions, with the latter arising from the treatment of molecular vibrations, rotations, and translations at finite temperatures. The thermal contributions are typically calculated using the rigid-rotor harmonic oscillator approximation based on the vibrational frequencies and geometry from a density functional theory (DFT) calculation. Several factors can introduce significant errors in the thermal free energies: (1) the accuracy of the DFT functional in predicting vibrational frequencies and the geometry, (2) the treatment of low-frequency modes where the harmonic approximation breaks down, (3) the neglect of the anharmonicity of the vibrational modes, and (4) the contribution of multiple conformers to the partition function.

In this study, we benchmark the thermal contributions to cluster binding free energies for atmospheric molecular dimers. We examine the performance of various DFT functionals and basis sets for calculating vibrational frequencies and thermal free energies using the quasi-harmonic approximation (QHA).^78^ Our test set comprises monomers and dimers of SA, FA, NA, MSA, AA, W, AM, MA, DMA, and TMA, with vibrational frequencies and geometries calculated at the DF-CCSD­(F12b)­(T*)/cc-pVDZ-F12 level of theory. Furthermore, we fit anharmonic scaling factors from VPT2 calculations to account for anharmonicity.

Finally, using the best found basis set and functional combinations along with the anharmonic scaling factors, we compare their performance to experimental free energies of complexes^79–83^ consisting of 2,2,2-trifluorethanol (TFE), TMA, dimethyl ether (DME), ethanol (EtOH), trimethylphosphine (TMP), methanol (MeOH), and dimethyl sulfide (DMS) with and without including multiconformer effects.^84^

## 2 Methodology

### 2.1 Computational Details

The DF-CCSD­(F12b)­(T*)/cc-pVDZ-F12 ^85,86^ vibrational frequency calculations were computed in MOLPRO^87,88^ using the default parameters.

The DFT geometry and harmonic frequency calculations with ωB97X-D3BJ,^89^ PW91,^90^ and M06-2X^91^ using the Pople style,^92–95^ Karlsruhe,^96,97^ and Jensen^98^ basis sets were done with ORCA 6.1.0 ^99–101^ as well as the composite B97-3c,^102^ r^2^SCAN-3c,^103^ and ωB97X-3c^104^ calculations. In all cases, TightSCF and the default auxiliary basis sets were employed. For M06-2X, the DEFGRID3 setting was employed.

ORCA 6.1.0 was employed for the final structures used for the VPT2 calculations, where VeryTightOPT, ExtremeSCF, and *Z*_tol_ = 10^–14^ were utilized.

The LNO-CCSD­(T)^71–76,105^ calculations were performed using MRCC 25.1.2 with default settings.^106–108^

### 2.2 Free Energy Calculations

The binding free energy is defined as the free energy gain from bringing the monomers together from indefinitely apart

1 $$\Delta G=G_{dimer}-\sum_{i}^{N_{monomers}}G_{monomer}^{i}$$

where the sum runs over the number of monomers. Any term with a Δ in front, we define similarly as a binding term. The Gibbs free energy can be subdivided into an electronic part and a thermal part.

2 $$G=E+G_{thermal}$$

In this study, we benchmark the *G*_thermal_ term i.e., everything but the electronic single point, as we already benchmarked the optimal single point method.^77,109^ Of special interest are the zero point vibrational energy contribution to the thermal free energy

3 $$ZPE=\frac{1}{2}hc\sum_{i}^{N_{vibrations}}v_{i},$$

where *N*_vibrations_ is the number of vibrational modes, *h* is the Planck constant, *c* the speed of light, and *v* the vibrational frequency. The vibrational entropy contribution to the thermal free energy, which in the harmonic approximation is given as

4 $$S_{vib}^{harm}=R\sum_{i}^{N_{vibrations}}[\frac{hcv_{i}}{k_{B}T\left(\exp\left(\frac{hcv_{i}}{k_{B}T}\right)-1\right)}-\ln\left(1-\exp\left(-\frac{hcv_{i}}{k_{B}T}\right)\right)],$$

where *R* is the gas-constant.

A problem with the harmonic entropy contribution, is that for shallow potentials, several low vibrational frequencies appear and cause the logarithmic term to tend toward infinity, yielding a nonphysical large entropic stabilization. We employ the QHA,^78^ where the vibrational entropy contribution is calculated as a linear combination of a free rotor contribution *S*_rot,qh_ and the standard harmonic term *S*_vib_^harm^

5 $$S_{vib}^{qh}=w\left(v\right)S_{vib}^{harm}+\left(1-w\left(v\right)\right)S_{rot,qh}$$

The scaling function *w*(*v*) is given as

6 $$w\left(v\right)=\frac{1}{1+{\left(v_{0}/v\right)}^{4}},$$

where *v*_0_ is the QHA threshold. The threshold defines at which vibrational frequency the contribution is described as half rotational and half vibrational. The free rotor contribution is given as

7 $$S_{rot,qh}=\frac{1}{2}R+R\;\ln[{\left(\frac{8\pi^{3}\mu^{\prime }k_{B}T}{h^{2}}\right)}^{1/2}].$$

μ′ is the reduced average molecular moment of inertia given as

8 $$\mu^{\prime }=\frac{\mu I_{avg}}{\mu +I_{avg}}$$

The multiconformer entropy correction to the binding free energy global minimum is calculated according to Partanen et al.^84^ as

9 $$\Delta \Delta G=-RT\;\ln\sum_{i}^{N_{confomers}}\exp\left(\frac{-\left(\Delta G_{i}-\Delta G_{0}\right)}{RT}\right),$$

where Δ*G*_*i*_ is the binding free energy of the *i*’th conformer and Δ*G*_0_ is the binding free energy of the global minimum conformer.

### 2.3 Calculating New Particle Formation Rates

NPF rates can be calculated from QC data by setting up a system of birth–death equations

10 $$\frac{dc_{i}}{dt}=\sum_{i=1}^{\left\lfloor i/2\right\rfloor }s_{j,\left(i-j\right)}c_{j}c_{\left(i-j\right)}+\sum_{j}\gamma_{\left(i+j\right)\;\to \;i}c_{i+j}-\sum_{j}s_{i,j}c_{i}c_{j}-\sum_{j}^{\left\lfloor i/2\right\rfloor }\gamma_{i\;\to \;j}c_{i},$$

where *c*_*i*_ is the concentration of cluster *i*, *t* is the time, γ_*i* → *j*_ is the evaporation coefficient of *i* to form *j*, and *s*_*i*,*j*_ is the sticking coefficient for sticking-collisions between *i* and *j*.

Assuming detail-balanced, the evaporation coefficient is given as

11 $$\gamma_{\left(i+j\right)\;\to \;i}=s_{ij}\frac{c_{i}^{e}c_{j}^{e}}{c_{i+j}^{e}}=s_{ij}c_{ref}\;\exp\left(\frac{\Delta G_{i+j}-\Delta G_{i\;}-\Delta G_{j}}{k_{B}T}\right),$$

where *c* ^e^ is the equilibrium concentration, *c*_ref_ the reference vapor concentration, Δ*G* the binding free energy, *T* the temperature, and *k*_B_ the Boltzmann constant. This is the term that causes the rate to be exponentially dependent on the binding free energies.

From these equations, errors in binding free energies can be used to estimate errors in NPF rates as exp­(Δ*G*_error_/(*RT*)), under the assumption the error is entirely determined by the evaporation rate of the specific cluster that gave the error.

### 2.4 The Reference Free Energies

We used the optimized geometries, calculated at the DF-CCSD­(F12b)­(T*)/cc-pVDZ-F12 level of theory, by Jensen and Elm^110^ as the starting point for the frequency calculations. Schmitz and Christiansen^111^ showed that the resulting frequencies are fairly converged toward the CBS limit, as the DF-CCSD­(F12b)­(T*)/cc-pVDZ-F12 frequencies have a root-mean-square deviation of 6 cm^–1^ to the CCSD­(F12)­(T*)/cc-pVQZ-F12 reference.

Due to the high memory requirements of the calculations, we were only able to converge the following dimers and their monomers.

#### Acid–Acid Dimers

(NA)_2_, (FA)_2_, (AA)_2_, (NA)_1_(FA)_1_, (NA)_1_(AA)_1_, (SA)_1_(FA)_1_, (SA)_1_(NA)_1_, (MSA)_1_(FA)_1_, and (FA)_1_(AA)_1_.

#### Acid–water Dimers

(SA)_1_(W)_1_, (NA)_1_(W)_1_, (MSA)_1_(W)_1_, (FA)_1_(W)_1_, and (AA)_1_(W)_1_.

#### Acid–base Dimers

(SA)_1_(MA)_1_, (SA)_1_(AM)_1_, (NA)_1_(AM)_1_, (NA)_1_(MA)_1_, (NA)_1_(DMA)_1_, (MSA)_1_(MA)_1_, (MSA)_1_(AM)_1_, (FA)_1_(TMA)_1_, (FA)_1_(DMA)_1_, (FA)_1_(AM)_1_, (FA)_1_(MA)_1_, (AA)_1_(MA)_1_, and (AA)_1_(AM)_1_. These cover the known important atmospheric molecular cluster formation interactions of inorganic acid–water, inorganic acid−ammonia and inorganic acid-alkylamines. It also covers the same interactions with organics through AA and FA, although actual organic nucleation likely requires multifunctional organic compounds to occur.

The free energy calculations use a quasi-harmonic threshold of 100 cm^–1^ as suggested by Grimme^78^ and to be consistent with the standard behavior of the ORCA program.^100^ The effect of changing the threshold on atmospheric molecular clusters has been tested by Elm et al.^112^ They found a difference between the choice of cutoff is below 1.7 and 1.4 kcal/mol for the (SA)_1–4_(DMA)_1–4_ and (SA)_1–4_­(putrescine)_1–4_ cluster systems, respectively, using thresholds between 100–200 cm^–1^. However, the size of the correction scales mostly with the size of the clusters, indicating reaction free energies, and in extension, evaporation rates, stay consistent within the threshold range. If a method predicts a binding thermal free energy that is too high compared to the reference we will define it as “underbinding” whereas a prediction that is too low is “overbinding”.

### 2.5 Cluster Sampling Workflow

For the sampling of the hydrogen bonded complexes, ABCluster^113–115^ was used to generate initial conformers of the dimers using a population of 3000, 200 generations, a scout limit of 4, and saving 1000 local minima.^116,117^ All 1000 structures were then optimized, and had their vibrational frequencies computed at the targeted DFT level. A modified version of ArbAlign^118^ was then used to remove identical conformers based on a root-mean-square-deviation (RMSD) cutoff of 0.38 Å as suggested by Kildgaard et al.^119,120^ All unique conformers subsequently had a single point computed at the Normal LNO–CCSD­(T)/aug-cc-pVTZ and Normal LNO–CCSD­(T)/aug-cc-pVQZ level of theory for a two-point extrapolation^121,122^ to the CBS limit, with the extrapolation coefficients suggested by Neese and Valeev.^123^

## 3 Results and Discussion

### 3.1 Benchmark of Thermal Free Energies

The exponential dependence on the free energy (eq 1) requires the thermal energy term to be as accurate as possible. However, it is not computationally feasible to run high accuracy CCSD­(T) geometry optimizations and vibrational frequency calculations on all the relevant clusters. In this section, we test the ability of different combinations of functionals and basis sets ability to match the Δ*G*_thermal_, *T*Δ*S*, and binding ZPE terms. A summary table of the MAEs, and number of imaginary conformers for the given method is available in the section S5.

Unfortunately, default settings for the optimization in ORCA lead to some clusters having small imaginary modes. These imaginary modes are small rotations of the clusters rather than a transition state, and according to our reference calculations, they should all be real. Because the specific clusters that end up having an imaginary mode is functional and basis set dependent, we therefore make the assumption that if only one imaginary mode is present and its frequency is down to −50 cm^–1^, it can be flipped when calculating the thermal properties as suggested by Bursch et al.^124^ We furthermore find this practice acceptable, as the flipped frequencies are very small in magnitude and therefore mainly described by the QHA approximation.

Testing this approach for two cases at the B97-3c and ωB97X-D3BJ/def2-SVP level of theory by optimizing the structure with the “DEFGRID3” setting (See Supporting Information 3 for more information) we get agreement for the quasi-harmonic (100 cm^–1^) vibrational terms within 0.12 kcal/mol (≈23% error in the NPF rates).

In our data set, the flipped frequency conformer never ends up being the global minimum, thus it does not affect the binding free energies in our test set. However, it will affect the number of unique conformers entering the multiconformer sum or the benchmark data set.

### 3.1.1 Pople Style Basis Sets

The most commonly applied basis set for atmospheric molecular clusters is the Pople style basis sets,^92–95^ as they have been shown to offer a good cost-to-accuracy ratio for similarly sized basis sets.^125^ Figure 1 shows the error in the Δ*G*_thermal_, *T*Δ*S*, and binding ZPE terms, for the given functional using the Pople style basis sets, relative to the DF-CCSD­(F12b)­(T*)/cc-pVDZ-F12 reference.

**Figure 1:**
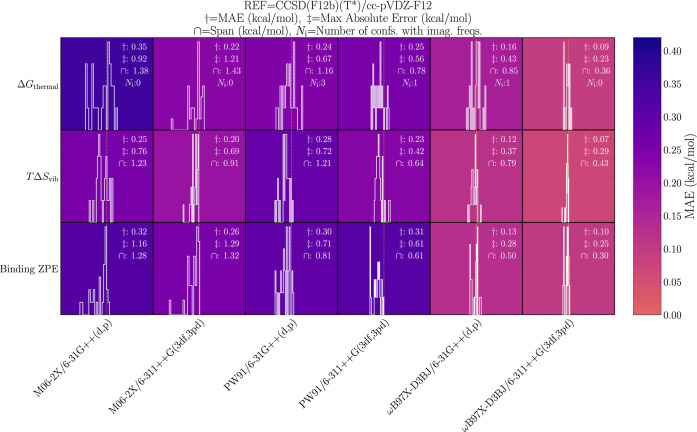
Error in Δ*G*_thermal_, *T*Δ*S*_vib_, and binding ZPE terms compared to the DF-CCSD­(F12b)­(T*)/cc-pVDZ-F12 reference values. Each box spans 4 kcal/mol. Negative values (left) indicate overbinding relative to the reference method. Free energies are calculated using the QHA with a threshold of 100 cm^–1^ and with imaginary modes flipped to real. *N*_*i*_ is the number of conformers where an imaginary mode is present for the given method.

Starting with the M06-2X functional, both basis set sizes exhibit no clusters with imaginary modes, likely due to the larger grid; however, they exhibit large errors with an outlier in Δ*G*_thermal_ of −0.92 kcal/mol [(MSA)_1_(MA)_1_] for the 6-31G++(d,p) basis set and −1.21 kcal/mol [(MSA)_1_(MA)_1_] for the 6-311++G­(3df,3pd) basis set. The M06-2X functional also overbinds the binding ZPE and *T*Δ*S*_vib_ terms for almost all the clusters.

Comparing the PW91 functional to the M06-2X functional, PW91 exhibits a smaller MAE in Δ*G*_thermal_ for the 6-31G++(d,p) basis (0.24 vs 0.35 kcal/mol) but 3 of the conformers contain imaginary modes. These appear in the (FA)_1_(MA)_1_, (MSA)_1_(MA)_1_, and (NA)_2_ clusters, and all exhibit small imaginary modes corresponding to rotations between the two monomers.

For the 6-311G­(3df,3dp) basis set, the MAE is actually larger (0.25 vs 0.22 kcal/mol) but the number of conformers with imaginary modes is reduced, with only the imaginary mode in [(FA)_1_(MA)_1_] remaining. Similar to the M06-2X functional, PW91 also overbinds the binding ZPE and *T*Δ*S*_vib_ in most cases.

The ωB97X-D3BJ functional performs the best of the three functionals, with the lowest MAE among them (0.16 kcal/mol for 6-31G++(d,p) and 0.09 kcal/mol for 6-311++G­(3df,3pd)). The largest errors are also significantly lower at −0.43 kcal/mol for the (MSA)_1_(FA)_1_ cluster using 6-31G++(d,p) and −0.23 kcal/mol for (SA)_1_(FA)_1_ cluster using the 6-311++G­(3df,3pd) basis set. While it still overbinds the *T*Δ*S*_vib_ and binding ZPE terms, the maximum errors are almost half of those of the other functionals for the same basis set. Unfortunately, the smaller basis set still predicts that a single dimer, (MSA)_1_(FA)_1_, has an imaginary mode. It again corresponds to the rotation between the two monomers.

Our findings indicate that the common choice of using the ωB97X-D/6-31G++(d,p) level of theory (which Jensen et al.^109^ showed gives the same result as the D3BJ dispersion) yields reliable thermal free energies. The MAE error corresponds to an ≈31% error in the NPF rate and the maximum error can yield a NPF rate that is wrong by a factor of 2. These could potentially be improved by using the larger Pople basis set if the target clusters are small enough. However, errors from neglecting anharmonicity and multiconformer entropy effects are likely much larger than these differences.

A general trend we also observe for other basis sets is that the ωB97X-D3BJ functional outperforms the other functionals. Therefore, we will focus entirely on it in the two following sections. However, we have added the equivalent figures for the PW91 and M06-2X functionals in the Supporting Information S1.

### 3.1.2 Karlsruhe Basis Sets

The Karlsruhe basis sets^96,97^ have also been widely applied due to their parametrization for a large part of the periodic system and their inherent design to systematically converge toward the basis set limit with increasing size. Figure 2 shows the error in the Δ*G*_thermal_, *T*Δ*S*, and binding ZPE terms for the ωB97X-D3BJ functional using the Karlsruhe style basis sets, with and without minimal augmentation, relative to the DF-CCSD­(F12b)­(T*)/cc-pVDZ-F12 reference.

**Figure 2:**
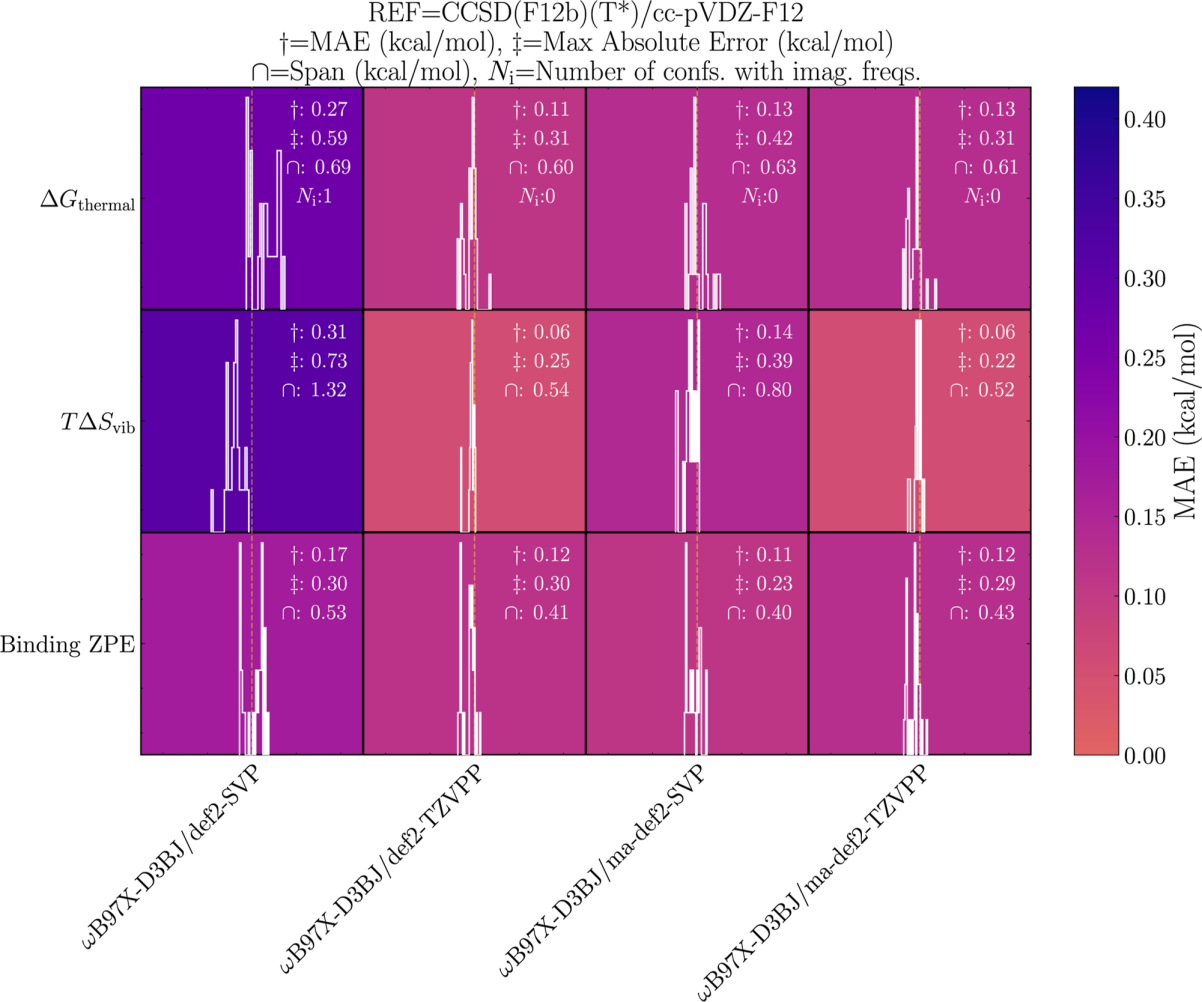
Error in Δ*G*_thermal_, *T*Δ*S*_vib_, and binding ZPE terms compared to the DF-CCSD­(F12b)­(T*)/cc-pVDZ-F12 reference values. Each box spans 4 kcal/mol. Negative values (left) indicate overbinding relative to the reference method. Free energies are calculated using the QHA with a threshold of 100 cm^–1^ and with imaginary modes flipped to real. *N*_*i*_ is the number of conformers where an imaginary mode is present for the given method.

The Karlsruhe basis sets perform quite well, with the lowest MAE being for the def2-TZVPP basis set at 0.11 kcal/mol and the largest MAE being the def2-SVP basis set at 0.27 kcal/mol. The minimal augmented double-ζ basis set beats the double-ζ Pople basis set, but the Pople triple-ζ basis still has a lower MAE than both the Karlsruhe triple-ζ basis sets. Similarly to the Pople basis, only the smallest basis set contains a cluster with an imaginary mode. This is the (AA)_1_(MA)_1_ cluster, where the mode again corresponds to an internal rotation between the monomers.

Similar to before, we also see that the *T*Δ*S*_vib_ term overbinds, except for the largest basis set (ma-def2-TZVPP), where the error distribution is more centered around zero. Adding the diffusion functions significantly improves the def-SVP results, almost halving the MAE for all terms. However, adding it for the def2-TZVPP basis set, makes no difference in the MAE of the *T*Δ*S*_vib_ and binding ZPE terms and actually increases the MAE by 0.02 kcal/mol for the Δ*G*_thermal_ term. This suggests that the remaining discrepancies are likely not originating from the basis set error.

The lower MAE in Δ*G*_thermal_ and the absence of imaginary modes make the ωB97X-D3BJ/ma-def2-SVP level of theory a better alternative for calculating the thermal contributions compared to the commonly applied ωB97X-D3BJ/6-31++G­(d,p) level of theory. The MAE error corresponds to an ≈25% error in the NPF rate and the maximum error can yield a NPF rate that is wrong by a factor of 2. However, for higher accuracy applications, the triple-ζ Pople basis set is still superior.

### 3.1.3 Jensen Basis Sets

Another set of basis sets that are designed to systematically converge toward the basis set limit for DFT calculations are the polarizable consistent (pc) basis sets by Jensen.^98^ Unfortunately, there does not exist a “minimal” augmented version of the pcseg basis set. We previously found that the fully augmented basis sets lead to numerical problems due to linear dependencies for larger atmospheric molecular clusters.^126^ We therefore removed the augmented basis functions on hydrogen to ensure more numerical stability (as done before on atmospherically relevant systems^77,127^ ). Basis sets where this has been done are denoted as aug’. Figure 2 shows the error in the Δ*G*_thermal_, *T*Δ*S*, and binding ZPE terms, for the ωB97X-D3BJ functional using the Jensen style basis sets, with and without augmentation, relative to the DF-CCSD­(F12b)­(T*)/cc-pVDZ-F12 reference. With the adjusted basis set, all the pcseg calculations are relatively well-behaved with a low MAE in Δ*G*_thermal_ of 0.19, 0.13, 0.15, and 0.14 kcal/mol for the pcseq-1, pcseq-2, aug’-pcseg-1, and aug’-pcseg-2 basis sets, respectively. The pcseg-1 basis set is the only one that exhibits a conformer [(MSA)_1_(FA)_1_] with an imaginary mode. The aug’-pcseg-1 is almost as good as the ma-def2-SVP basis set in terms of MAE but exhibits a bit larger MAE (0.15 vs 0.13 kcal/mol) in Δ*G*_thermal_ and the binding ZPE term (0.13 vs 0.11 kcal/mol). However, it is better for the *T*Δ*S*_vib_ term with a MAE of 0.10 kcal/mol compared to 0.14 kcal/mol.

While the aug’-pcseg-1 basis does not beat the ma-def2-SVP basis set, it is an improvement on def2-SVP. This suggests that if a minimal augmented version of pcseg is created, it might outperform the basis sets we have tested so far. Similarly to before, for higher accuracy applications, the triple-ζ Pople basis set is still superior.

### 3.1.4 Methods with Intrinsic Basis Sets

Alternatively to functionals where the basis set is user-specified the empirical corrected 3c series of methods by the Grimme group^102–104^ come with intrinsic basis sets that have been specifically optimized to lower the error with the given functional and empirical corrections. The r^2^SCAN-3c, B97-3c, and ωB97X-3c methods have previously been explored for atmospheric molecular clusters. Especially, B97-3c has shown an excellent compromise between accuracy and cost for larger molecular clusters.^128^

Figure 4 shows the error in the Δ*G*_thermal_, *T*Δ*S*, and binding ZPE terms, for the ωB97X-3c, B97-3c, and r^2^SCAN-3c composite methods, relative to the DF-CCSD­(F12b)­(T*)/cc-pVDZ-F12 reference. Of the three composite methods, ωB97X-3c performs the best, but it is also the most costly method. Comparing it to the best functional and basis set combination, ωB97X-D3BJ/ma-def2-SVP, it has a higher MAE in Δ*G*_thermal_ (0.15 vs 0.13 kcal/mol) but a lower span (0.30 vs 0.42 kcal/mol) and max absolute error (0.30 vs 0.42 kcal/mol). The lower max absolute error also decreases the possible error in the NPF rate from a factor of 2 to a factor 1.66. It performs much better for the *T*Δ*S*_vib_ term with almost half the span and MAE of the ωB97X-D3BJ/ma-def2-SVP level of theory, while again being outperformed in the binding ZPE term. The ωB97X-3c level of theory also predicts the (SA)_1_(MA)_1_ to have a single imaginary mode, which like in all other cases, corresponds to rotation between the monomers. The results are also consistent with the findings by Wu et al.^129^

**Figure 3:**
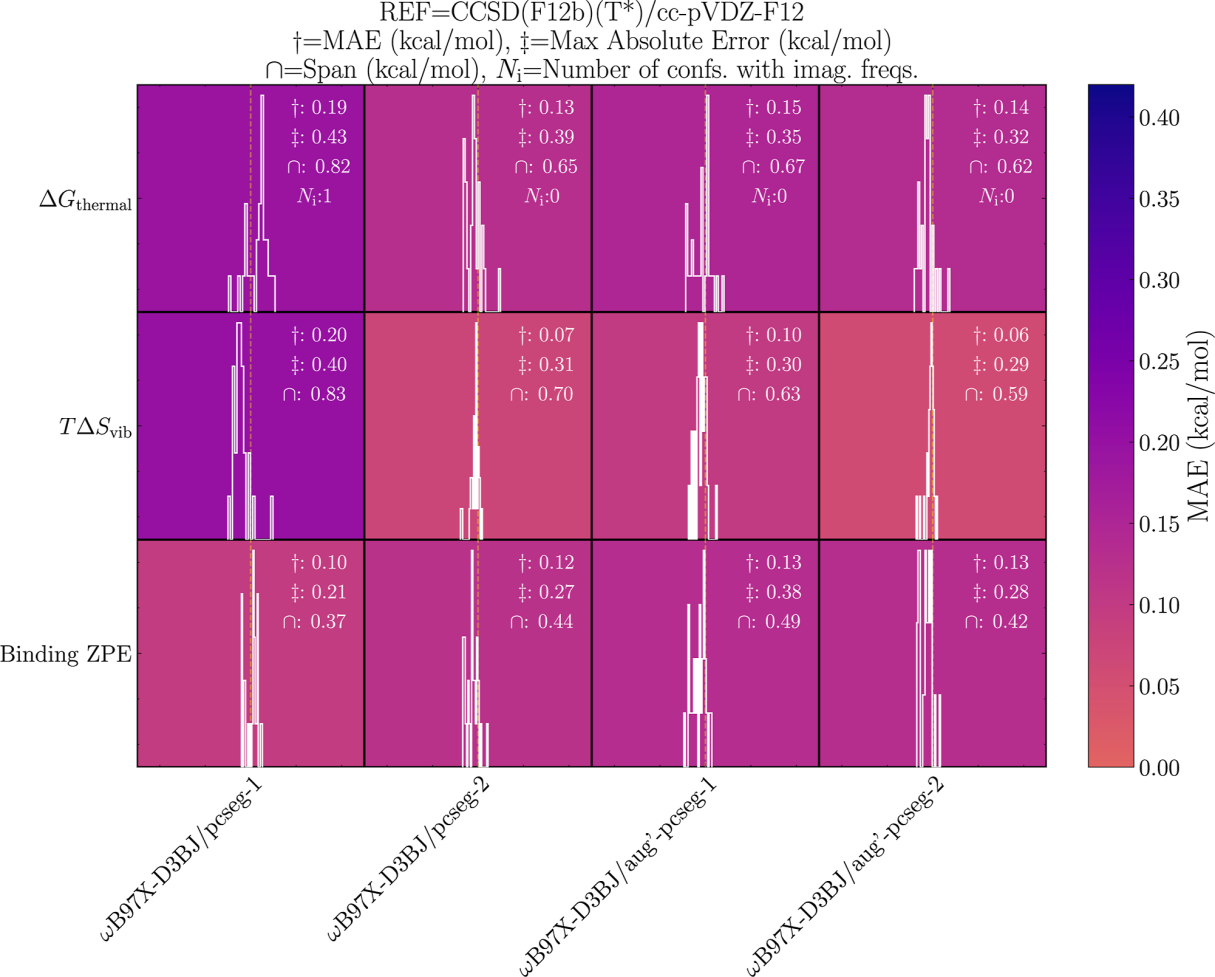
Error in Δ*G*_thermal_, *T*Δ*S*_vib_, and binding ZPE terms compared to the DF-CCSD­(F12b)­(T*)/cc-pVDZ-F12 reference values. Each box spans 4 kcal/mol. Negative values (left) indicate overbinding relative to the reference method. Free energies are calculated using the QHA with a threshold of 100 cm^–1^ and with imaginary modes flipped to real. *N*_*i*_ is the number of conformers where an imaginary mode is present for the given method.

**Figure 4:**
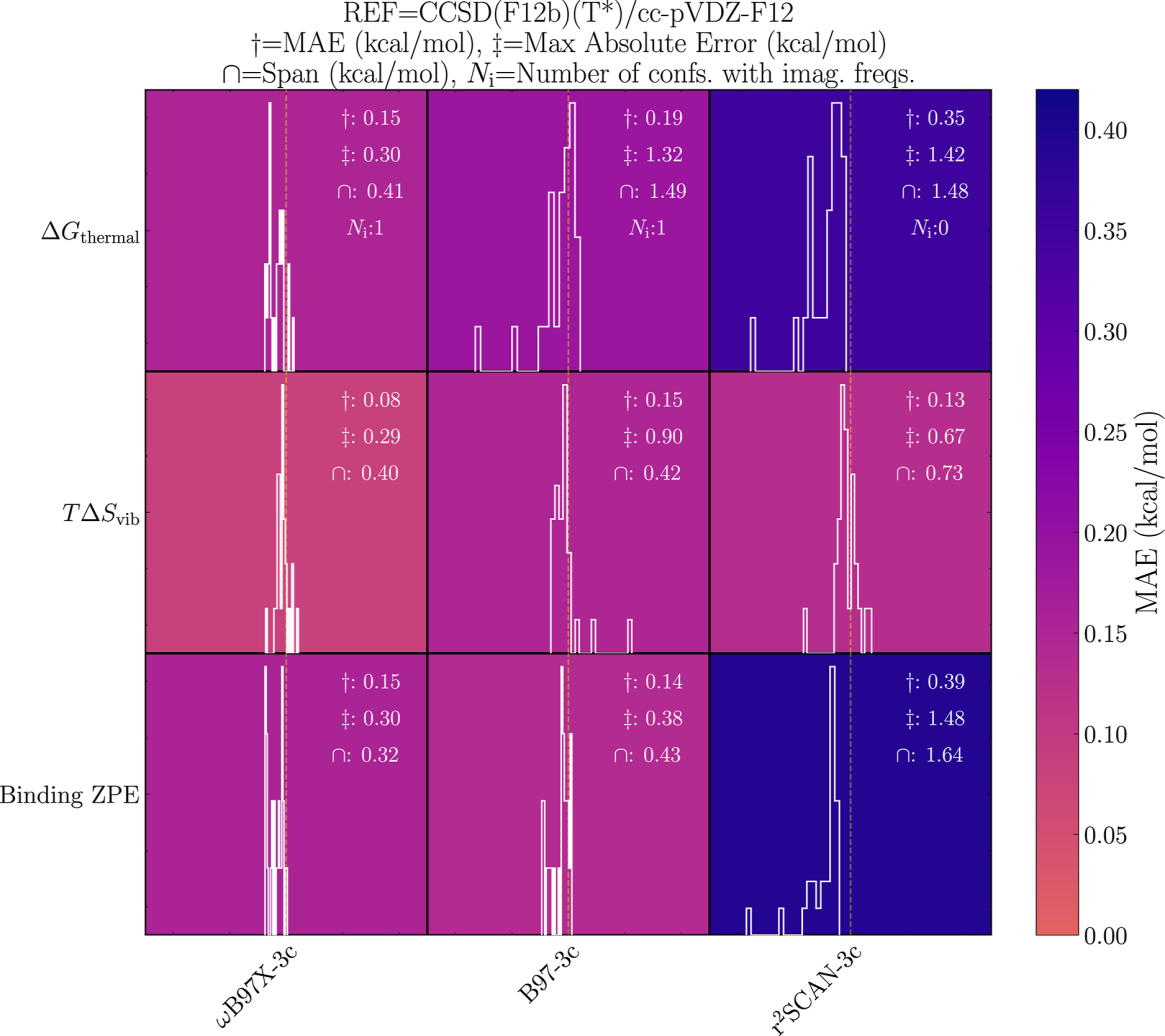
Error in Δ*G*_thermal_, *T*Δ*S*_vib_, and binding ZPE terms compared to the DF-CCSD­(F12b)­(T*)/cc-pVDZ-F12 reference values. Each box spans 4 kcal/mol. Negative values (left) indicate overbinding relative to the reference method. Free energies are calculated using the QHA with a threshold of 100 cm^–1^ and with imaginary modes flipped to real. *N*_*i*_ is the number of conformers where an imaginary mode is present for the given method.

Comparing the cheaper methods, both exhibit outliers in the Δ*G*_thermal_ term, but B97-3c has a significantly lower MAE of 0.19 kcal/mol (≈37% error in the NPF rate) compared to r^2^SCAN-3c with 0.35 kcal/mol (≈80% error in the NPF rate). This is due to r^2^SCAN-3c overbinding compared to the reference method, whereas B97-3c errors are more centered on zero. r^2^SCAN-3c also shows significant outliers in the binding ZPE term, −1.47 kcal/mol for (MSA)_1_(MA)_1_ (a factor of 12 error in the NPF rate) and −0.99 kcal/mol (a factor of 5 error in the NPF rate) for (SA)_1_(MA)_1_, which do not appear for the B97-3c results. These results agree with previous benchmark studies that show that r^2^SCAN-3c performs worse than B97-3c for atmospheric clusters.^129,130^ However, unlike r^2^SCAN-3c, B97-3c predicts the (SA)_1_(MA) dimer to have an imaginary mode. For larger molecular clusters, such as freshly nucleated particles,^128^ only the cheaper methods are applicable. This is due to the introduction of Fock-exchange in range-separated hybrid functionals which increases the scaling exponent by one (see the timing differences between B97-3c and ωB97X-3c on Figure 3 in Müller et al.^104^). Therefore, we recommend the B97-3c method, for the calculation of thermal contributions, as it exhibits lower MAEs compared to the PW91 functional and the r^2^SCAN-3c level of theory and it does not exhibit outliers in the binding ZPE term. This is furthermore based on benchmarks by Wu et al.,^129^ Engsvang et al.^131^ who showed the energies and geometries are decent for larger clusters, but a single-point energy correction should still be applied.^77^

### 3.2 Anharmonic Scaling Factors

One of the major approximations when calculating the free energy is that the vibrations can be described as harmonic. A common strategy within the computational chemistry field is finding anharmonic scaling factors, such that the computed free energies match experimental measurements.^132^ This approach ends up correcting more than just the harmonic approximation, as it also fits differences from the method, the basis set error and multiconformer effects. However, this approach is not applicable for atmospheric molecular clusters, as experimental measurements are scarce. Temelso et al.^133^ suggested using the VPT2 ^134^ method to calculate anharmonic frequencies for the clusters and then using these to fit anharmonic scaling factors that are used within the standard harmonic partition function. We used a single scaling factor as Neefjes et al.^66^ found no significant differences in the correction when using a split-region approach.

We followed this approach to calculate anharmonic scaling factors for the ωB97X-D3BJ/ma-def2-SVP and B97-3c level of theories on the data set. Due to the numerical third derivatives, the VPT2 calculations can be numerically unstable. If the calculations end up giving imaginary anharmonic modes, the dimer or monomer was discarded from the fit. Likewise, we plotted the frequencies and anharmonic frequencies against each other and dimers or monomers containing obvious outliers were also removed from the fit. This process left 8 structures (114 frequencies) for the ωB97X-D3BJ/ma-def2-SVP fit and 17 structures (396 frequencies) for the B97-3c fit.

The harmonic frequencies as a function of the anharmonic frequencies, along with the scaling factor given by the fit and the *R* ^2^ value are shown in Figure 5.

**Figure 5:**
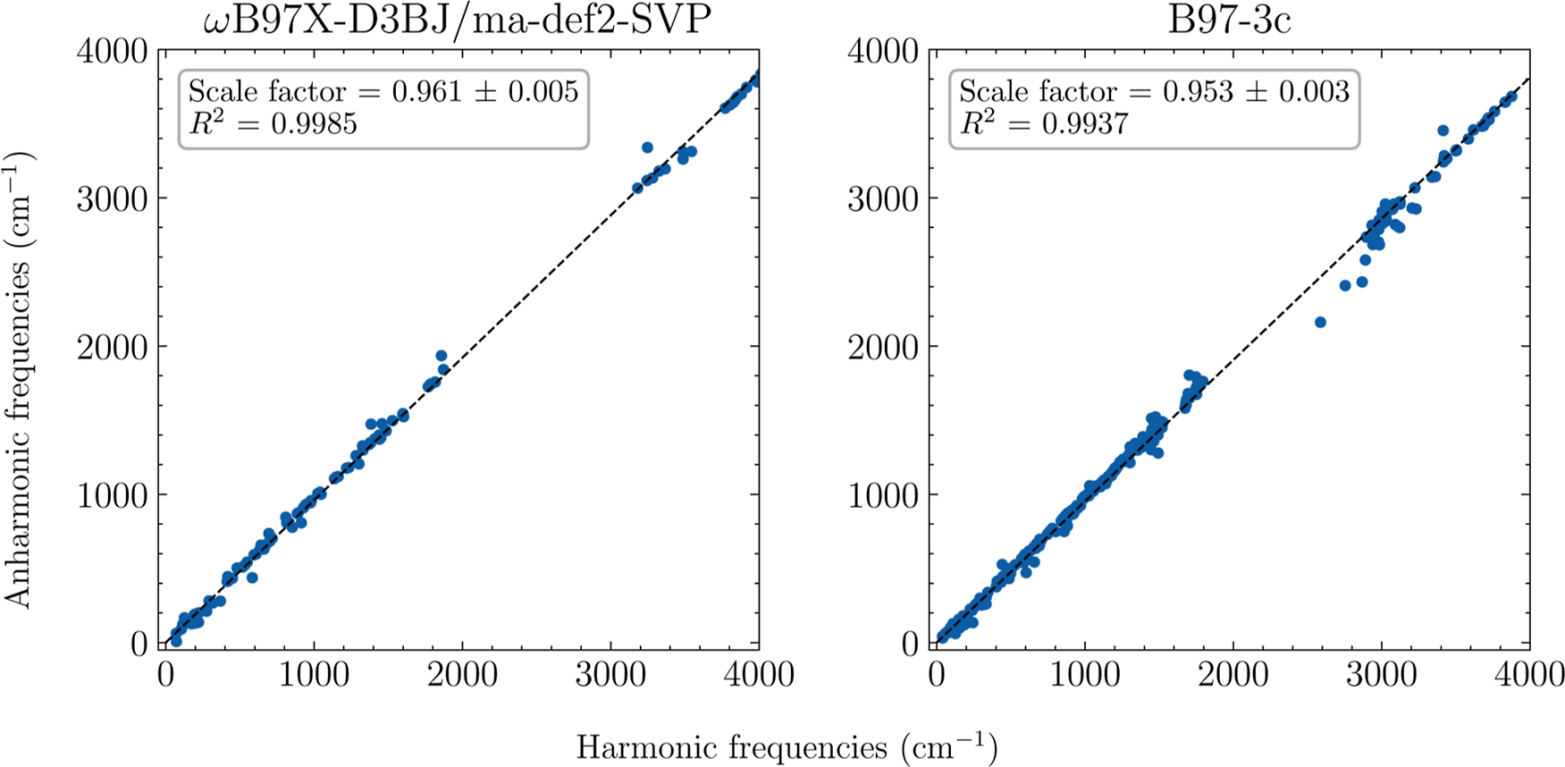
Harmonic frequencies plotted against anharmonic frequencies calculated with VPT2 using ωB97X-D3BJ/ma-def2-SVP and B97-3c. The fitted scale factor and *R* ^2^ value are shown in the boxes. Eight structures (114 frequencies) was used for the ωB97X-D3BJ/ma-def2-SVP fit and 17 structures (396 frequencies) for the B97-3c fit. The uncertainty represents the 95% confidence interval.

As seen in the figure, we obtain a scaling factor of 0.961 and 0.953 for the ωB97X-D3BJ/ma-def2-SVP and B97-3c frequencies, respectively. Both also have high *R* ^2^ values of 0.9985 and 0.9937 respectively. The slightly better fit for the ωB97X-D3BJ/ma-def2-SVP data set is likely due to fewer data points. In both cases, we obtain similar results to Neefjes et al.^66^ who got 0.944 for B97-3c and 0.950 for the similar ωB97X-D/6-31++G­(d,p) level of theory, when calculating anharmonic scaling factors using VPT2 on a set of dry/hydrated (acid and/or base)_0–2_(W)_0–5_ clusters consisting of SA, AM, MA, DMA, and MSA. The derived scaling factor also generally falls within the range of other studies^125,132,135^ despite applying different underlying methods, different data sets, and different filtering rules. The effect of the scaling factor on dimers is also minimized by the QHA approximation, for instance, a scaling factor of 0.95 versus 0.96 gives sub 0.05 kcal/mol differences (≈9% error in the NPF rate). Hence, for these two reasons, we consider these scaling factors applicable outside the fitted data set on similar noncovalent bounded dimers.

### 3.3 Matching Experimental Free Energies

As stated before, experimental measurements of atmospheric molecular clusters are scarce. In the review by Hansen et al.^79^ they collected free energy estimations of a number of hydrogen-bonded bimolecular complexes^80–83^ using a combination of gas phase infrared spectroscopy and vibrational theory. The complexes consist of 2,2,2-trifluorethanol (TFE), TMA, dimethyl ether (DME), ethanol (EtOH), trimethylphosphine (TMP), methanol (MeOH), and dimethyl sulfide (DMS) and are depicted in Figure 6.

**Figure 6:**
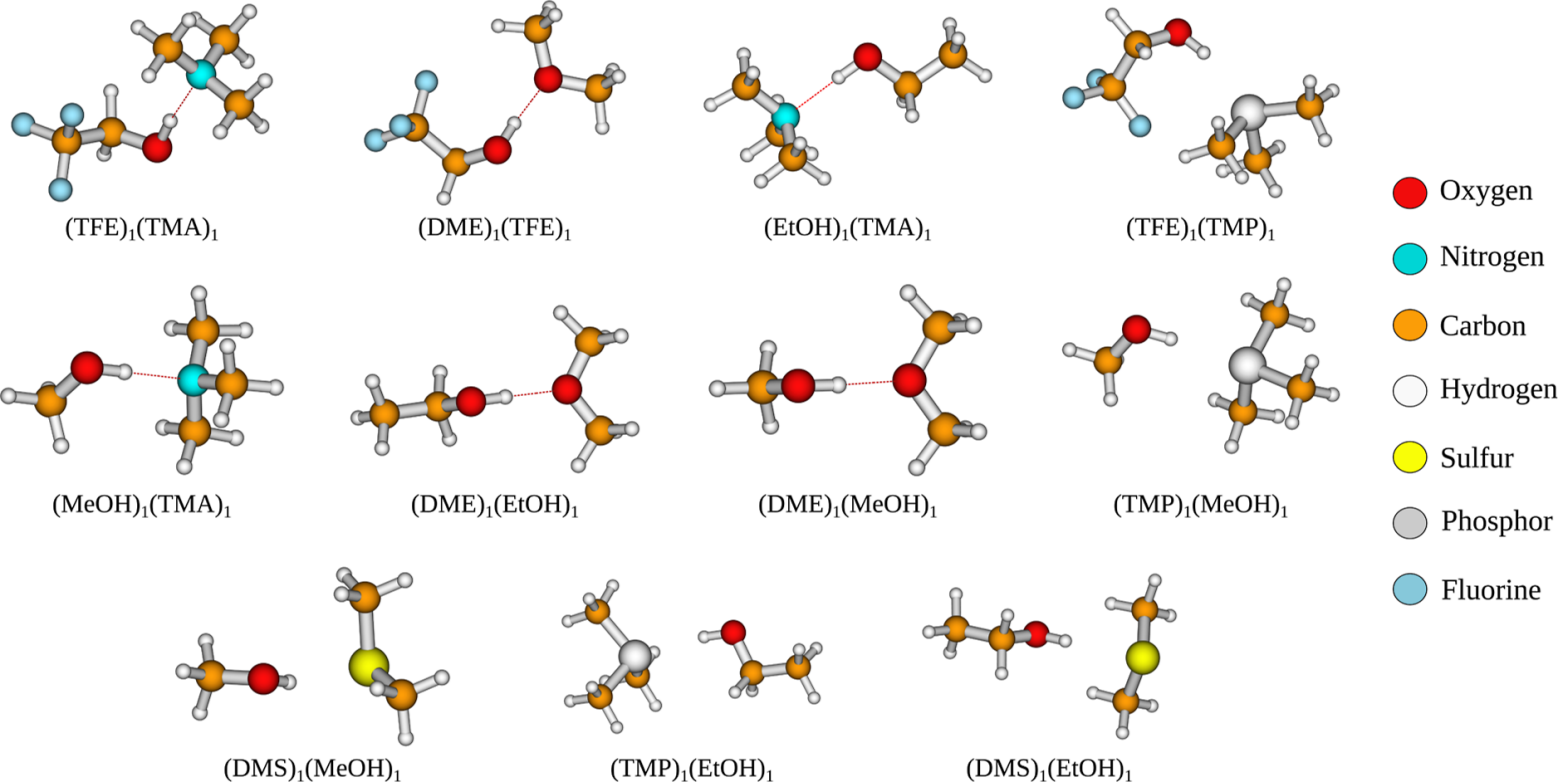
Hydrogen-bonded bimolecular complexes for which experimental free energies are available. Atom types are given by the side label.

While these are not the same molecules as before, the dimers span similar intermolecular interactions and were chosen by the authors to be atmospherically relevant.^79^ For instance, the O–H–O, O–H–S, and O–H–N interactions are present, which correspond to the major interactions driving organic–organic and organic–inorganic cluster growth. While the SA–base interaction is not directly present. DMS, which is the precursor to SA and MSA, is present and the nitrogen-based base TMA is present. We therefore believe the data set to be a useful test case for validation. Hence, if our workflow also works for these, it shows the robustness of the underlying methodology.

Figure 7 shows the error in the calculated quasi-harmonic (threshold at 100 cm^–1^ binding free energy at the Normal LNO–CCSD­(T)/CBS­(aug’-3,aug’-4)//ωB97X-D3BJ/ma-def2-SVP level of theory using an anharmonic scaling factor of 0.961. We tested both with or without including multiconformer effects. For cases where the conformers yielded imaginary frequencies, the conformers were either removed or the mode was flipped.

**Figure 7:**
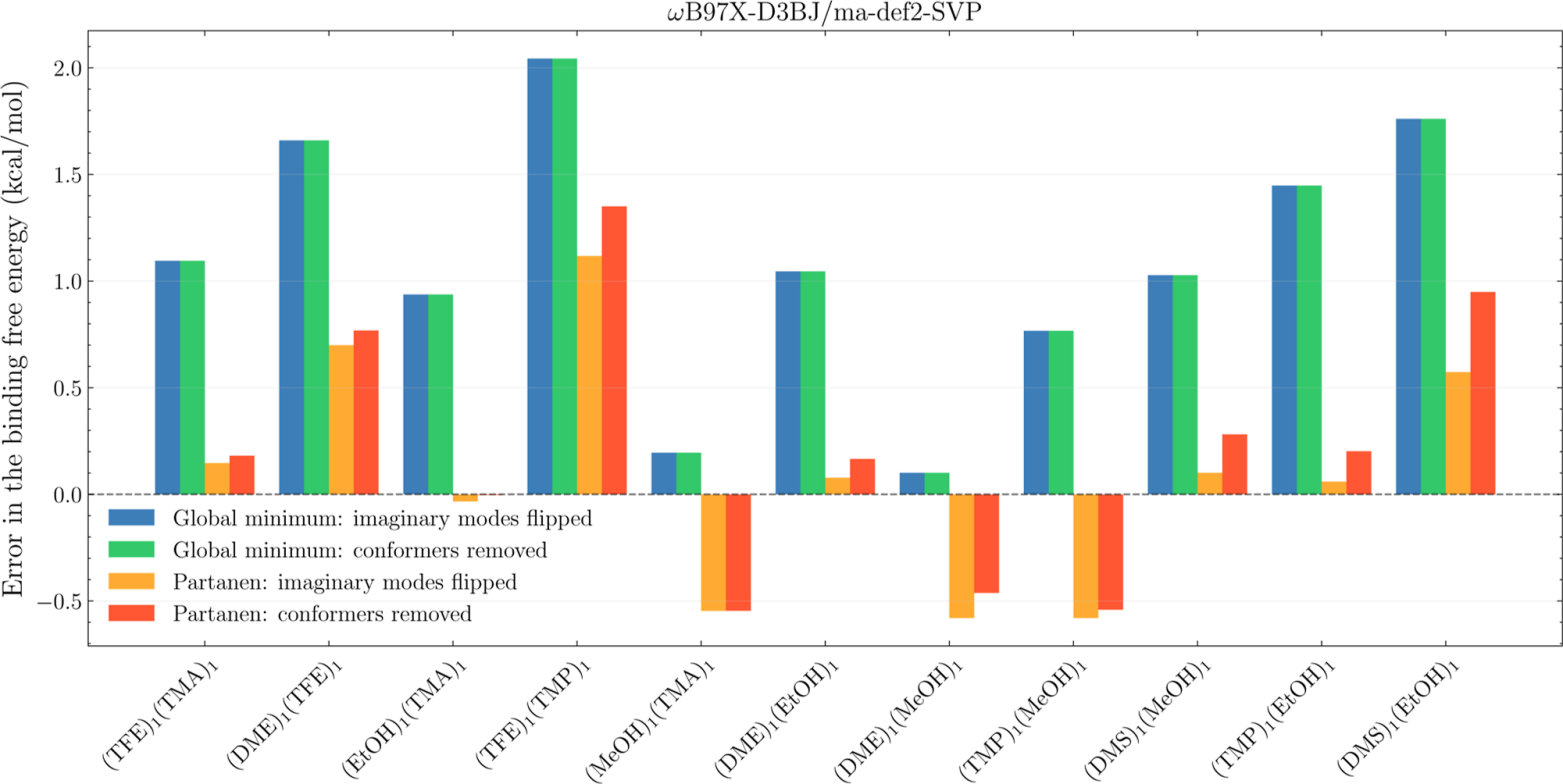
Error in the binding free energy compared to the reference when taking the global free energy minimum where conformers with imaginary modes have been flipped (blue), global free energy minimum where conformers with imaginary modes have been removed (green), multiconformer free energy where conformers with imaginary modes have been flipped (orange) or multiconformer free energy where conformers with imaginary modes have been removed (red). All calculations are at the Normal LNO–CCSD­(T)/CBS­(aug’-3,aug’-4)//ωB97X-D3BJ/ma-def2-SVP using the QHA approximation with a threshold of 100 cm^–1^ and a anharmonic scaling factor of 0.961.

The single-conformer free energy approach systematically underbinds most clusters relative to the reference values. Including multiconformer effects significantly improves accuracy: the MAE drops from 1.1 kcal/mol (a factor of 6.4 error in the NPF rate) to 0.43 kcal/mol (a factor of 2 error in the NPF rate) when imaginary modes are flipped, and to 0.50 kcal/mol (a factor of 2.3 error in the NPF rate) when conformers with imaginary modes are removed. The flipped approach performs better because it retains more conformers, leading to a larger Partanen sum correction that compensates for the underbinding observed with the global minimum approach.

However, overcorrection occurs for some systems where the global minimum approach already performs well. For the (MeOH)_1_(TMA)_1_ and (DME)_1_(MeOH)_1_ dimers, the global minimum values are already within 0.2 kcal/mol of the reference, and the Partanen sum then overcorrects, increasing the error. A similar pattern appears for the (TMP)_1_(MeOH)_1_ cluster, where the global minimum error of roughly 0.8 kcal/mol becomes roughly −0.5 kcal/mol after applying the multiconformer correction.

Despite these cases of overcorrection, the multiconformer approach achieves maximum errors below 1 kcal/mol for nearly all clusters. The only exception is the (TFE)_1_(TMP)_1_ cluster, which shows an error of roughly 1.1 kcal/mol when imaginary modes are flipped. This represents a substantial improvement over the global minimum approach, which has a maximum error of up to 2.0 kcal/mol. It should be noted that the results are highly sensitive to the conformer uniqueness criteria, as changes to the number of retained conformers can significantly alter the magnitude of the multiconformer correction. For instance, switching the RMSD threshold from 0.38 Å to 1.03 Å for the (DME)_1_(EtOH)_1_ systems reduced the number of conformers from 17 to 6, yielding a change of 0.66 kcal/mol in the multiconformer binding free energy (see Section S4 for the sensitivity analysis). We therefore believe determining a robust conformer uniqueness criteria is an important next step of study. Although not present in our current tests, for clusters containing large flexible organic molecules, erroneously labeling identical conformers as unique would result in a significant overcounting of the configurational entropy. The systems are also sensitive to the QHA threshold: changing it from 100 cm^–1^ to 0 cm^–1^ (i.e., purely harmonic) without scaling lowers the binding free energy by −2.2 kcal/mol to −3.6 kcal/mol, which is larger in magnitude than the binding free energies of the references themselves. The large difference is due to noncovalent bounded molecules having several low frequency modes giving an artificially large entropic stabilization. In this case, the stabilization is so large that the pure harmonic approximation, with no scaling, yields binding free energies that are too negative in all cases compared to the experimental values (0.5 to 2.7 kcal/mol too low). It would therefore never be possible to match the experimental values using the harmonic approximation with the current methodology, since the multiconformer and anharmonic effects would further stabilize the clusters.

The error in the calculated quasi-harmonic (threshold at 100 cm^–1^) binding free energy at the Normal LNO–CCSD­(T)/CBS­(aug’-3,aug’-4)//B97-3c level of theory using an anharmonic scaling factor of 0.953, with or without including multiconformer effects for the cases where conformers with imaginary modes are either removed or have been flipped compared to the reference, is shown in Figure 8.

**Figure 8:**
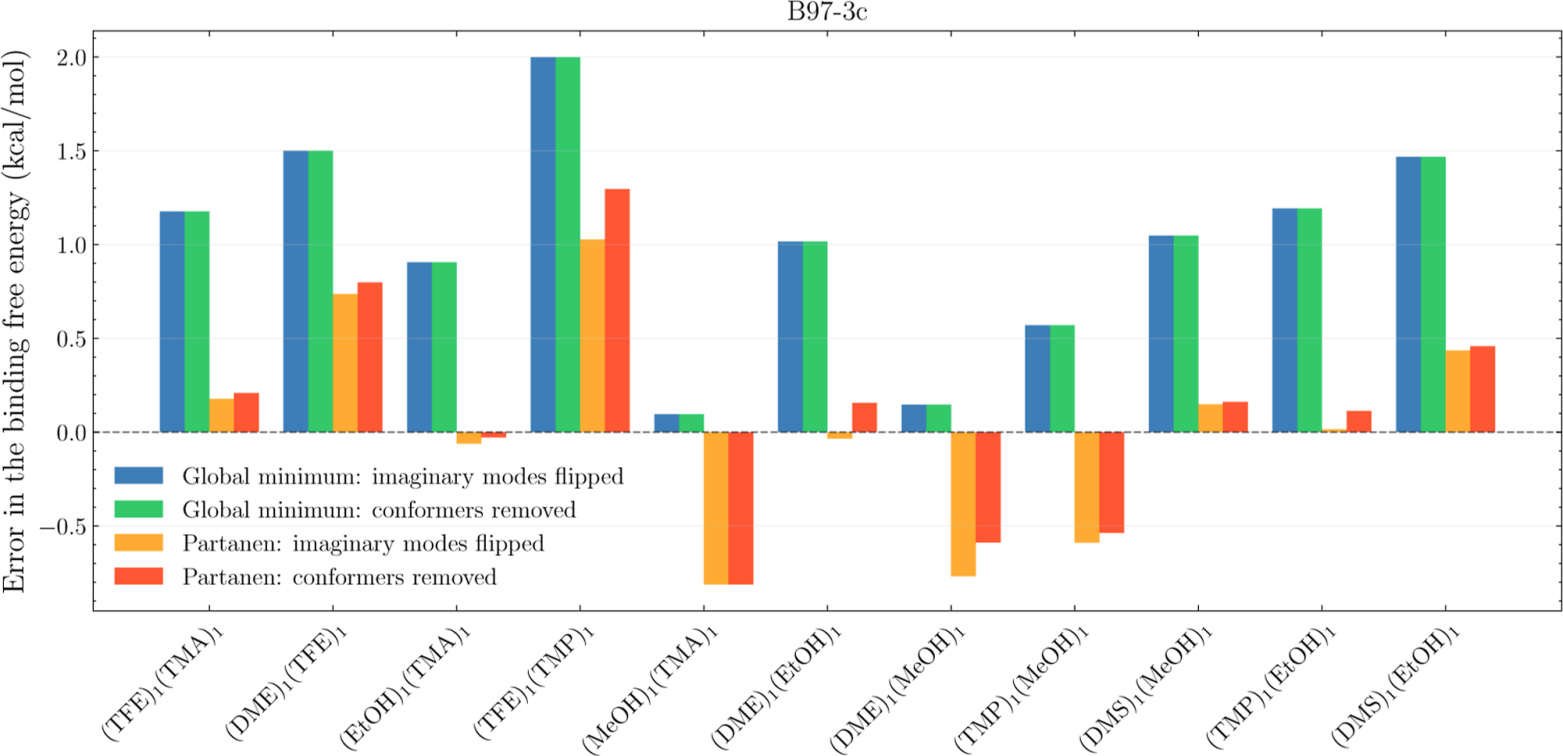
Error in the binding free energy compared to the reference when taking the global free energy minimum where conformers with imaginary modes have been flipped (blue), global free energy minimum where conformers with imaginary modes have been removed (green), multiconformer free energy where conformers with imaginary modes have been flipped (orange), or multiconformer free energy where conformers with imaginary modes have been removed (red). All calculations are at the Normal LNO–CCSD­(T)/CBS­(aug’-3,aug’-4)//B97-3c using the QHA approximation with a threshold of 100 cm^–1^ and a anharmonic scaling factor of 0.953.

The global minimum errors are within 0.29 kcal/mol (≈60% difference in predicted NPF rates) of the LNO–CCSD­(T)/CBS­(aug’-3,aug’-4)//ωB97X-D3BJ/ma-def2-SVP errors. The MAE and maximum error for the global minimum approach are actually around 0.1 kcal/mol lower compared to ωB97X-D3BJ/ma-def2-SVP, demonstrating that B97-3c is an excellent cost-to-accuracy method. This also indicates that the major difference between the two methods is the number of unique conformers entering the Partanen sum. However, the trends remain consistent across both methods, only the magnitude of the errors changes. Overall, the results indicate that the largest sources of error in cluster binding free energies, compared to the experimental values, are multiconformer effects and the treatment of vibrational frequencies. If the error behavior is similar to other noncovalently bound systems, current binding free energies are likely overestimated when low vibrational modes are treated using the harmonic approximation, i.e., the clusters are overbound. However, they are likely underestimated if the QHA with a threshold of 100 cm^–1^ is applied. However, the error can be lowered when multiconformer effects and an anharmonic scaling factor is applied. We therefore recommend using anharmonic vibrational scaling factors in the range of 0.94–0.96. If high-accuracy binding free energies are needed, a more specific factor should be fitted for the applied method.

We recommend using the QHA approach to treat the low vibrational modes, as we would not be able to match the experimental free energies with a pure harmonic approach. The major assumption here is what to set the threshold to. We recommend using 100 cm^–1^ as suggested by Grimme^78^ and to be consistent with the ORCA program.

We also recommend including multiconformer effects. However, establishing a robust criterion for conformer uniqueness remains an open challenge beyond the scope of this study. Once such a criterion has been determined, a specific QHA threshold should be fitted for the final alignment to experimental data.

## 4 Conclusions

We have benchmarked the thermal contributions of binding free energies for atmospheric molecular clusters by computing reference harmonic frequencies and quasi-harmonic free energies at the DF-CCSD­(F12b)­(T*)/cc-pVDZ-F12 level of theory for 11 monomers and 27 dimers relevant to atmospheric cluster formation. Our assessment of DFT functionals (M06-2X, PW91, ωB97X-D3BJ) combined with Pople style, Karlsruhe, and Jensen basis sets, along with composite methods (B97-3c, r^2^SCAN-3c, ωB97X-3c), shows that the ωB97X-D3BJ/ma-def2-SVP level of theory provides the best balance of accuracy and efficiency. It has a mean absolute error (MAE) of 0.13 kcal/mol for the thermal free energy and showed no imaginary modes for any of the clusters in the test set.

For larger molecular clusters, such as freshly nucleated particles, we recommend the B97-3c composite method as it offers an excellent compromise between computational cost and accuracy without a large increase in MAE on the dimer testset. This is consistent with the results by Wu et al.^129^

VPT2 calculations were performed to determine anharmonic scaling factors of 0.961 and 0.953 for ωB97X-D3BJ/ma-def2-SVP and B97-3c frequencies, respectively, which are in agreement with previous studies.

Finally, we tested our approach against experimental dimer free energies of hydrogen-bonded complexes and showed that incorporating anharmonic scaling factors, together with multiconformer entropy effects and a high-level Normal LNO–CCSD­(T)/CBS­(aug-3,aug-4) single-point correction yields binding free energies with errors below 1 kcal/mol compared to the reference.

Our results indicate that the largest sources of error in the quasi-harmonic binding free energies of our atmospheric molecular dimer test set stem from the neglect of multiconformer contributions and, to a lesser extent, the neglect of anharmonicity of the vibrational frequencies.

## Supplementary Material



## Notes

The benchmark results for the PW91 and M06-2X functionals with the Jensen and Karlsruhe basis sets Link to the structures and output files (PDF)
