# Supplementary material for: Coupled Cluster Free Energies for Atmospheric Molecular Clusters: Benchmark and Matching Experimental Free Energies
Source: ACS Omega. 2026 Jun 17;11(25):36893–907. doi: 10.1021/acsomega.6c00573 (PMC13325095; doi:10.1021/acsomega.6c00573)
Supplement: Supplementary file 1 [file ao6c00573_si_001.pdf]

Supporting Information: Coupled Cluster Free Energies for Atmospheric Molecular Clusters: Benchmark and Matching Experimental Free Energies

Yosef Knattrup, Andreas Buchgraitz Jensen, and Jonas Elm*

Department of Chemistry, Aarhus University, Langelandsgade 140, 8000 Aarhus C, Denmark

E-mail: jelm@chem.au.dk

Phone: +45 28938085

S1 Benchmark results for PW91 and M06-2X

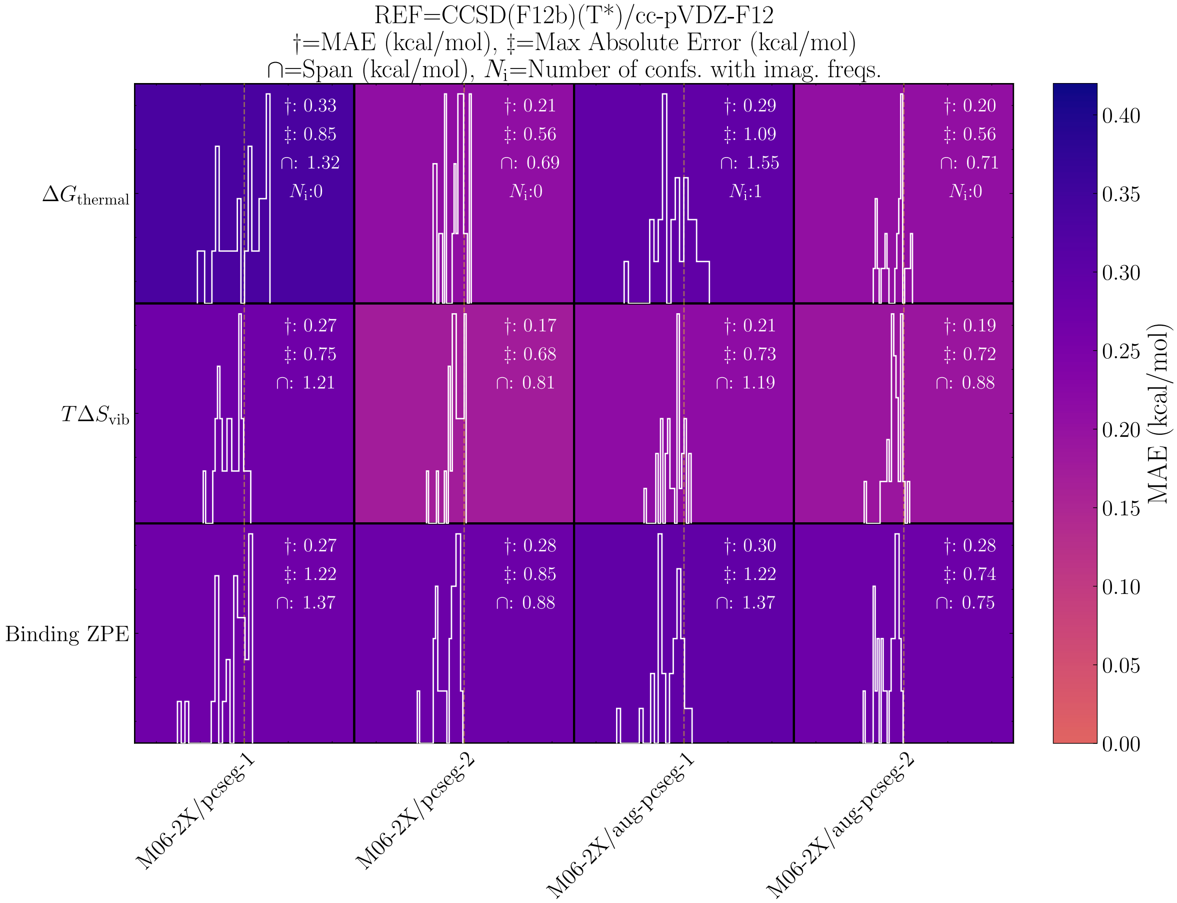

Figure S1: Error in $\Delta G_{\mathrm{thermal}}$, $T\Delta S_{\mathrm{vib}}$, and binding ZPE terms compared to the DF-CCSD(F12b)(T*)/cc-pVDZ-F12 reference values. Each box spans 4 kcal/mol. Negative values (left) indicate overbinding relative to the reference method. Free energies are calculated using the QHA with a threshold of 100 cm^{-1} and with imaginary modes flipped to real. N_i is the number of conformers where an imaginary modes is present for the given method.

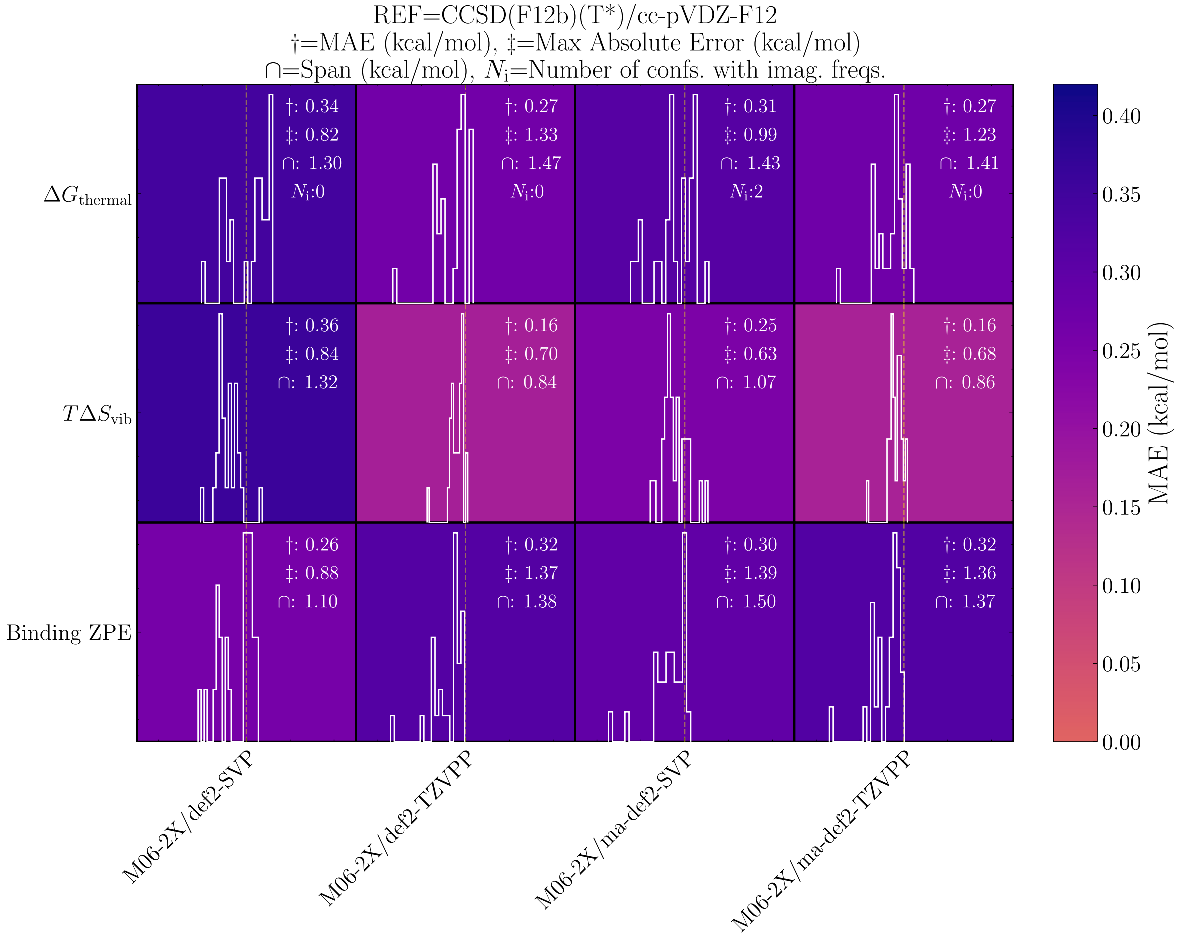

Figure S2: Error in $\Delta G_\mathrm{thermal}$, $T\Delta S_\mathrm{vib}$, and binding ZPE terms compared to the DF-CCSD(F12b)(T*)/cc-pVDZ-F12 reference values. Each box spans 4 kcal/mol. Negative values (left) indicate overbinding relative to the reference method. Free energies are calculated using the QHA with a threshold of 100 cm^{-1} and with imaginary modes flipped to real. N_i is the number of conformers where an imaginary modes is present for the given method.

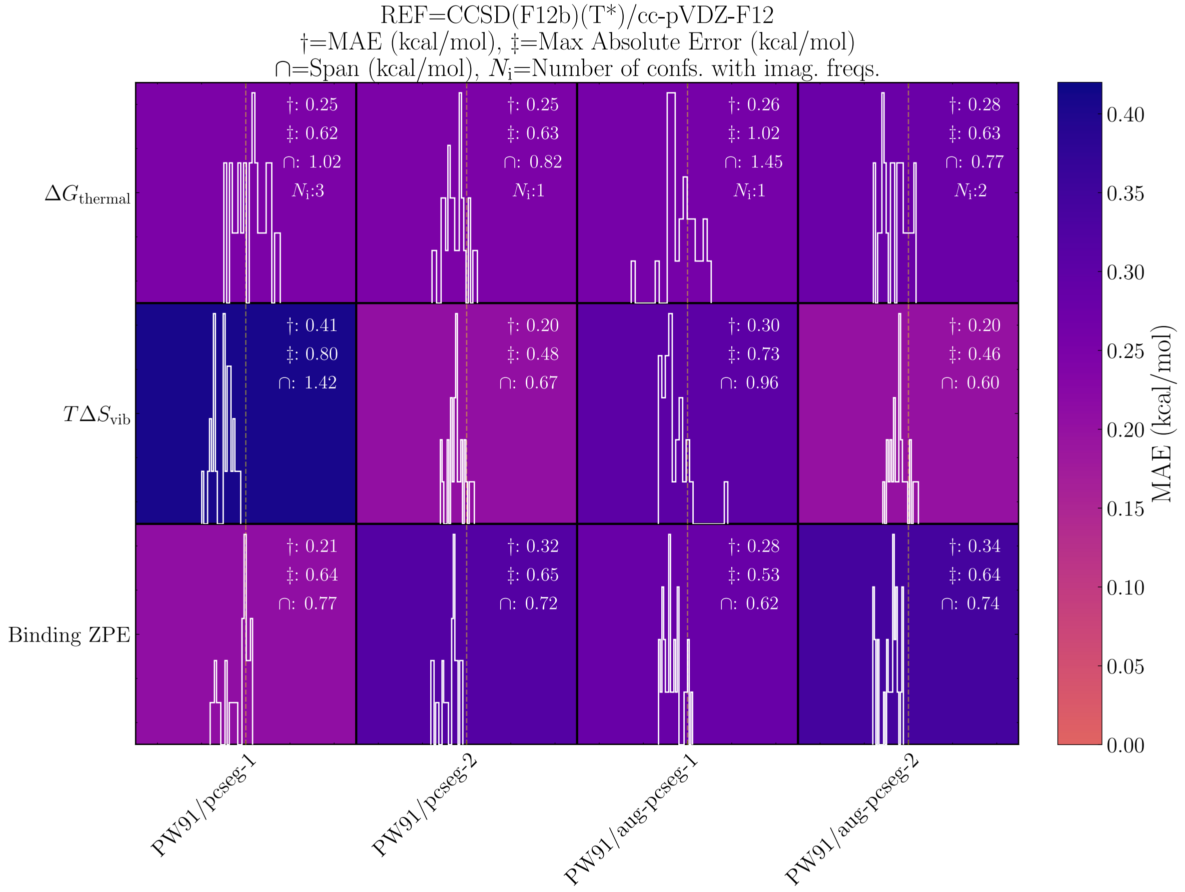

Figure S3: Error in $\Delta G_\mathrm{thermal}$, $T\Delta S_\mathrm{vib}$, and binding ZPE terms compared to the DF-CCSD(F12b)(T*)/cc-pVDZ-F12 reference values. Each box spans 4 kcal/mol. Negative values (left) indicate overbinding relative to the reference method. Free energies are calculated using the QHA with a threshold of 100 cm^{-1} and with imaginary modes flipped to real. N_i is the number of conformers where an imaginary modes is present for the given method.

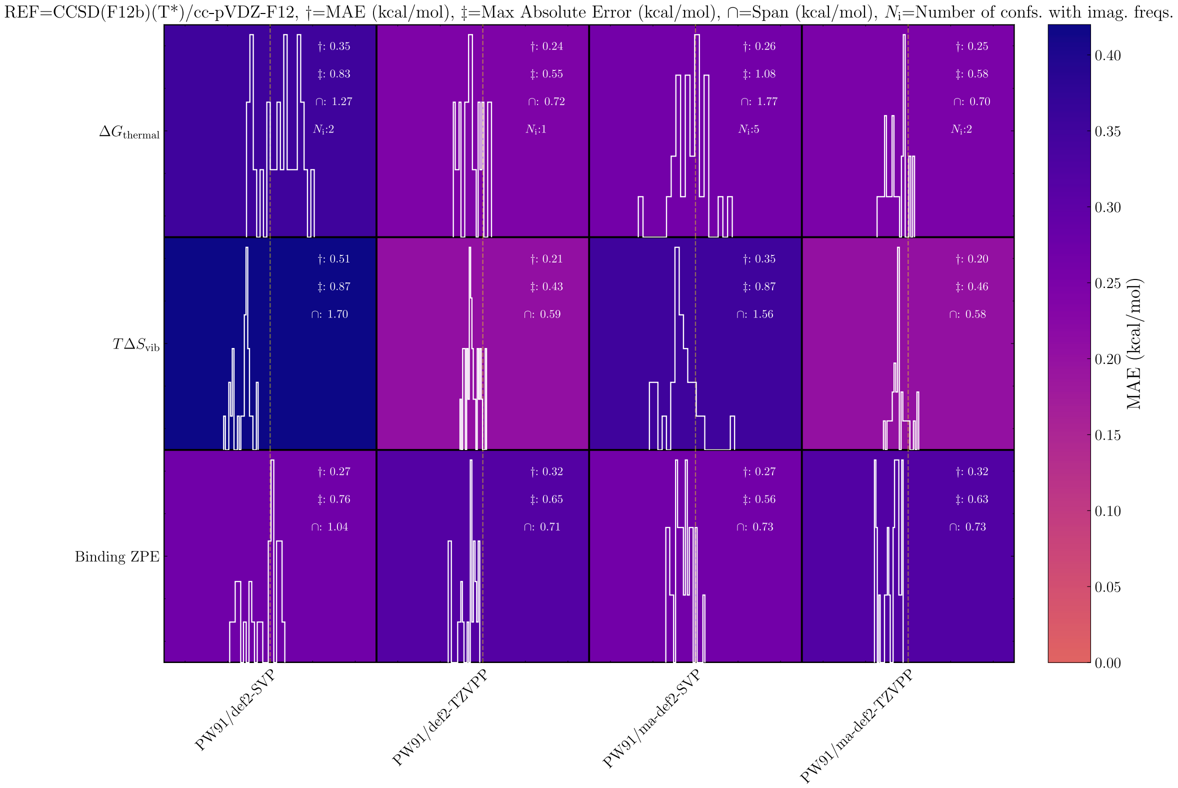

Figure S4: Error in $\Delta G_{\mathrm{thermal}}$, $T\Delta S_{\mathrm{vib}}$, and binding ZPE terms compared to the DF-CCSD(F12b)(T*)/cc-pVDZ-F12 reference values. Each box spans 4 kcal/mol. Negative values (left) indicate overbinding relative to the reference method. Free energies are calculated using the QHA with a threshold of 100 cm^{-1} and with imaginary modes flipped to real. N_i is the number of conformers where an imaginary modes is present for the given method.

S2 Data access

All the calculated properties and structures are available in the Atmospheric Cluster DataBase[1,2] at `https://github.com/elmjonas/ACDB/tree/master/Articles/knattrup26_free_energy_benchmark`

S3 Imaginary mode comparison

At the B97-3c level of theory, the $(NA)_1(AA)_1$ cluster contains an imaginary mode.
The frequencies (in cm^{-1}) are given as: -22.36, 40.06, 58.5, 109.76, 135.34, 176.39, 193.54, 455.73, 585.33, 613.67, 638.26, 686.48, 769.85, 839.21, 889.27, 939.31, 954.78, 1024.1, 1063.7, 1269.44, 1293.79, 1389.79, 1440.07, 1473.66, 1479.42, 1487.16, 1706.49, 1719.4, 2858.98, 3027.31, 3088.79, 3146.08, 3357.6.
The rotational constants (in cm^{-1}) are: 0.196581,0.029538, 0.025801
Optimizing the structure with DEFGRID3 gives the following frequencies (in cm^{-1}) : 15.39, 37.61, 59.97, 109.82, 135.17, 176.04, 193.37, 455.94, 584.94, 613.45, 638, 686.21, 769.73, 839.76, 889.12, 938.77, 955.65, 1023.97, 1063.83, 1269.02, 1293.62, 1389.94, 1439.91, 1473.74, 1479.22, 1487.34, 1706.36, 1719, 2858.58, 3027.34, 3088.61, 3146.34, 3359.49
and new rotational constants (in cm^{-1}): 0.196560, 0.029525, 0.025790.
Comparing the absolute value of the sum of the thermal vibrational energy, the zero-point energy, and the vibrational entropy, without the quasi-harmonic approximation, gives 68.7141637332309 and 68.9602220203953 kcal/mol for the flipped approach and the DEFGRID3 structure, respectively.
With the quasi-harmonic approximation using a threshold of 100 cm^{-1} they give 67.2805809842208 and 67.4036438043492 kcal/mol for the flipped approach and the DEFGRID3 structure, respectively.

At the level of theory, the ωB97X-D3BJ/def2-SVP cluster contains an imaginary mode. The frequencies (in cm^{-1}) are given as: -25.05, 61.55, 82, 126.15, 131.05, 203.37, 260.92, 443.96, 482.83, 602.81, 655.05, 911.2, 926.79, 1002.67, 1018.6, 1064.46, 1102.57, 1165.58, 1192.46, 1342.12, 1347.53, 1424.1, 1449.66, 1450.25, 1459.59, 1488.54, 1503.47, 1537.77, 1626.52, 1857.2, 2975.51, 3049.14, 3087.66, 3134.76, 3174.84, 3179.89, 3219.52, 3532.44, 3626.4

The rotational constants (in cm^{-1}) are: 0.255352, 0.051339, 0.047671

Optimizing the structure with DEFGRID3 gives the following frequencies (in cm^{-1}): 31.03, 66.47, 84.9, 121.04, 134.88, 197.02, 259.51, 446.89, 491.22, 603.41, 655.58, 917.16, 927.51, 1003.61, 1019.14, 1064.58, 1104.72, 1160.11, 1189.28, 1339.6, 1347.08, 1425.15, 1449.58, 1455.03, 1459.6, 1487.01, 1502.33, 1537.12, 1648.25, 1856.85, 2978.73, 3047.96, 3087.88, 3133.61, 3169.92, 3180.37, 3219.7, 3516.99, 3618.23

and new rotational constants (in cm^{-1}): 0.267715, 0.048612, 0.045009

Comparing the absolute value of the sum of the thermal vibrational energy, the zero-point energy, and the vibrational entropy, without the quasi-harmonic approximation, gives 93.2597444779493 and 93.0871042679478 kcal/mol for the flipped approach and the DEFGRID3 structure, respectively.

With the quasi-harmonic approximation using a threshold of 100 cm^{-1} they give 92.1815089220285 and 92.1154491516434 kcal/mol for the flipped approach and the DEFGRID3 structure, respectively.

S4 Multi-conformer Sensitivity Analysis

Figure S5 shows the multi-conformer binding free energy of the $(DME)_1(EtOH)_1$ system at the at the Normal LNO-CCSD(T)/CBS(aug'-3,aug'-4)//ωB97X-D3BJ/ma-def2-SVP using the QHA approximation with a threshold of 100 cm^{-1} and a anharmonic

scaling factor of 0.961. Different RMSD thresholds are tested as given by the x-axis.

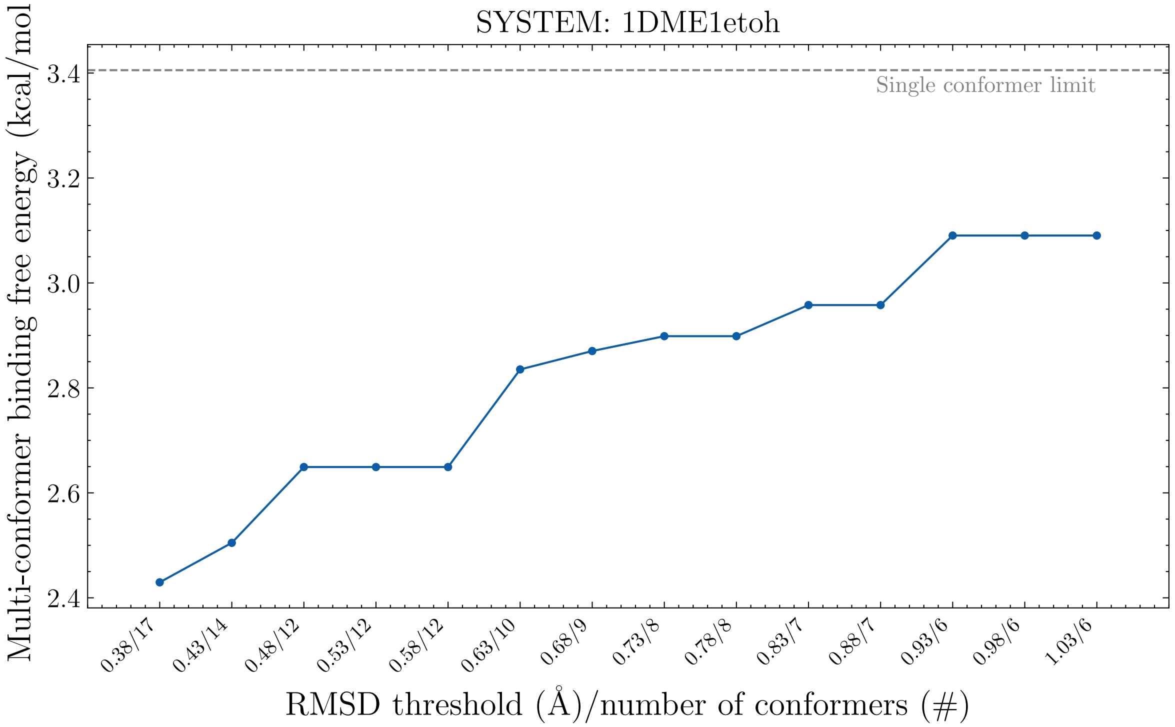

Figure S5: Multi-conformer binding free energy of the $(DME)_1(EtOH)_1$ system at the at the Normal LNO-CCSD(T)/CBS(aug'-3,aug'-4)//ωB97X-D3BJ/ma-def2-SVP using the QHA approximation with a threshold of 100 cm^{-1} and a anharmonic scaling factor of 0.961. Different RMSD threshold are tested as given by the x-axis.

S5 Summary of methods

Table S1: Statistical errors (values in kcal/mol)

**Method**	**MAE** $\Delta G_{\mathrm{thermal}}$	**MAE** $T\Delta S_{\mathrm{vib}}$	**MAE Binding ZPE**	**Imag. freqs.**
M06-2X/6-31G++(d,p)	0.35	0.25	0.32	0
M06-2X/6-311++G(3df,3pd)	0.22	0.20	0.26	0
PW91/6-31G++(d,p)	0.24	0.28	0.30	3
PW91/6-311++G(3df,3pd)	0.25	0.23	0.31	1
ωB97X-D3BJ/6-31G++(d,p)	0.16	0.12	0.13	1
ωB97X-D3BJ/6-311++G(3df,3pd)	0.09	0.07	0.10	0
ωB97X-D3BJ/pcseg-1	0.19	0.20	0.10	1
ωB97X-D3BJ/pcseg-2	0.13	0.07	0.12	0
ωB97X-D3BJ/aug'-pcseg-1	0.15	0.10	0.13	0
ωB97X-D3BJ/aug'-pcseg-2	0.14	0.06	0.13	0
ωB97X-D3BJ/def2-SVP	0.27	0.31	0.17	1
ωB97X-D3BJ/def2-TZVPP	0.11	0.06	0.12	0
ωB97X-D3BJ/ma-def2-SVP	0.13	0.14	0.11	0
ωB97X-D3BJ/ma-def2-TZVPP	0.13	0.06	0.12	0
ωB97X-3c	0.15	0.08	0.15	1
B97-3c	0.19	0.15	0.14	1
r^{2}SCAN-3c	0.35	0.13	0.39	0
PW91/pcseg-1	0.25	0.41	0.21	3
PW91/pcseg-2	0.25	0.20	0.32	1
PW91/aug-pcseg-1	0.26	0.30	0.28	1
PW91/aug-pcseg-2	0.28	0.20	0.34	2
M06-2X/pcseg-1	0.33	0.27	0.27	0
M06-2X/pcseg-2	0.21	0.17	0.28	0
M06-2X/aug-pcseg-1	0.29	0.21	0.30	1
M06-2X/aug-pcseg-2	0.20	0.19	0.28	0
PW91/def2-SVP	0.35	0.51	0.27	2
PW91/def2-TZVPP	0.24	0.21	0.32	1
PW91/ma-def2-SVP	0.26	0.35	0.27	5
PW91/ma-def2-TZVPP	0.25	0.20	0.32	2
M06-2X/def2-SVP	0.34	0.36	0.26	0
M06-2X/def2-TZVPP	0.27	0.16	0.32	0
M06-2X/ma-def2-SVP	0.31	0.25	0.30	2
M06-2X/ma-def2-TZVPP	0.27	0.16	0.32	0

References

(1) Elm, J. An Atmospheric Cluster Database Consisting of Sulfuric Acid, Bases, Organics, and Water. *ACS Omega* **2019**, *4*, 10965–10974.

(2) Kubečka, J.; Besel, V.; Neefjes, I.; Knattrup, Y.; Kurtén, T.; Vehkamäki, H.; Elm, J.

Computational Tools for Handling Molecular Clusters: Configurational Sampling, Storage, Analysis, and Machine Learning. *ACS Omega* **2023**, *8*, 45115–45128.
